# Creativity in Learning Analytics: A Systematic Literature Review

**DOI:** 10.3390/jintelligence13120153

**Published:** 2025-11-23

**Authors:** Siamak Mirzaei, Hooman Nikmehr, Sisi Liu, Fernando Marmolejo-Ramos

**Affiliations:** 1 UniSA Online, University of South Australia, Adelaide 5000, Australia; sisi.liu@unisa.edu.au; 2 UniSA STEM, University of South Australia, Mawson Lakes 5095, Australia; hooman.nikmehr@unisa.edu.au; 3 College of Education, Psychology and Social Work, Flinders University, Adelaide 5000, Australia; fernando.marmolejoramos@flinders.edu.au

**Keywords:** creativity, learning analytics, systematic review, educational technology, creativity metrics, data-driven learning

## Abstract

Creativity is increasingly recognized as an essential 21st-century skill, critical for innovation, problem-solving, and personal growth. Educational systems have responded by prioritizing creative thinking, prompting researchers to explore the potential of Learning Analytics (LA) to support and enhance creativity. This systematic review synthesizes empirical studies, theoretical frameworks, and methodological innovations from databases such as Web of Science, Scopus, ERIC, ProQuest, and Google Scholar, examining how creativity is operationalized within LA contexts. The review identifies diverse assessment frameworks, encompassing divergent thinking tests, product-based evaluations, behavioral metrics, and process-oriented assessments, often underpinned by the “4 Ps of Creativity” framework (Person, Process, Product, Press). Tools such as automated scoring systems, multimodal analytics, and AI-enhanced assessments demonstrate the potential to objectively and reliably capture creative processes and outcomes. However, significant challenges remain, including definitional ambiguity, inconsistent metrics, scalability issues, and ethical concerns related to data privacy. This review underscores the transformative capacity of LA to foster creativity in education while highlighting the critical need for standardized, robust methodologies and inclusive frameworks. By addressing identified gaps, future research can advance innovative approaches to assess and cultivate creativity using LA.

## 1. Introduction

Creativity has emerged as a critical skill for the 21st century, recognized as essential for problem-solving, innovation, and lifelong learning (OECD 2018). In education, cultivating creativity has become an increasingly prominent priority, reflecting its significance in fostering intellectual growth and adaptability (Venckutė et al. 2020). In the creativity literature, a common organizing view is the “4Ps” framework; Person, Process, Product, and Press, which clarifies that creativity involves learner dispositions/abilities, the cognitive–affective processes used, the qualities of outputs, and the learning environment (Henriksen et al. 2021). Creativity supports the ability to generate novel and valuable ideas, enabling individuals to adapt to ever-changing and complex environments (Henriksen et al. 2021). Concurrently, Learning Analytics (LA) has risen as a transformative tool in education, facilitating the analysis of learner data to improve teaching and learning outcomes (Ferguson 2012; Marrone and Cropley 2022). Recent work highlights how LA is evolving from descriptive dashboards toward multimodal and AI-assisted approaches that can capture process traces (e.g., interaction/discourse sequences) alongside product quality, offering new windows into creative thinking (Marrone and Cropley 2022).

The convergence of creativity and LA presents a unique opportunity to enhance educational practices. LA offers the ability to collect and analyze large-scale, multimodal data, providing educators with insights into how creativity emerges during learning processes (Hershkovitz et al. 2019). For instance, computational approaches have proven effective in measuring originality in programming (Chou et al. 2024) and analyzing creative outputs in platforms such as Scratch (Kovalkov et al. 2021). Framed against the 4Ps, these techniques make all four dimensions observable at scale, demonstrating LA’s potential to foster creative thinking through personalized feedback and adaptive learning environments.

Despite these advancements, several challenges remain. Defining and assessing creativity in educational settings is inherently complex, as creativity encompasses multiple dimensions, including individual traits (Person), cognitive processes (Process), tangible outputs (Product), and environmental factors (Press) (Li et al. 2022). Moreover, there is ongoing ambiguity about domain-general vs. domain-specific operationalizations and limited validation across contexts. Additionally, the integration of LA to support creativity often lacks standardized metrics and methodological consistency, along with ethical considerations (privacy, consent, and potential algorithmic bias), which pose obstacles to its scalability and generalizability (OECD 2018; Marrone and Cropley 2022).

The importance of nurturing creativity is echoed in international frameworks, such as the OECD Education 2030 project, which highlights creativity as a key skill for lifelong learning and societal innovation (OECD 2018). These policy signals underscore the need for valid and scalable approaches to monitoring and supporting creative thinking in classrooms and systems. Similarly, academic research continues to emphasize the role of creativity in developing students’ ability to think critically and adapt to emerging challenges (Venckutė et al. 2020). Yet, the intersection of creativity and LA remains an underexplored area, presenting an opportunity to address gaps in both theory and practice.

This systematic review aims to synthesize the current state of research at the intersection of creativity and LA, focusing on how LA tools can effectively capture, assess, and foster creativity within educational contexts. By identifying prevailing trends, challenges, and opportunities, this review aims to establish a foundation for future research and practical applications in creativity-driven LA. Specifically, we map how LA operationalizes creativity across the 4Ps, identify areas of construct and metric alignment, and surface open questions for responsible adoption (OECD 2018; Henriksen et al. 2021; Marrone and Cropley 2022), highlighting the need for robust methodologies and standardized metrics to advance the integration of creativity in LA-driven educational strategies.

## 2. Materials and Methods

The systematic review method is utilized in this study to gain insight into the existing body of literature and identify potential gaps in knowledge (Munn et al. 2018) related to creativity within the context of LA in educational settings given the interdisciplinarity and emerging nature of this field. Systematic reviews follow a structured approach to collect, appraise, and synthesize evidence, ensuring transparency, replicability, and comprehensive coverage of the topic under investigation (Gough et al. 2017). This methodology is particularly suited for exploring novel and interdisciplinary domains like creativity and LA, where diverse research methodologies and perspectives converge.

### 2.1. Project Team, Research Questions, and PICO Framework

A project team consisting of online and in-person educators from different disciplines conducted and collaborated on this systematic review. The team brought expertise in educational technology, creativity studies, and LA to ensure a comprehensive and interdisciplinary approach. The review was guided by the following research questions:

1. How are LA tools applied or developed to assess or foster creativity in educational settings?
2. What theoretical frameworks and methodologies are used when applying LA tools to study creativity in education?
3. What are the key challenges and limitations in integrating LA to support creativity in education?
4. What gaps exist in the current literature, and what future research directions can be identified?

This systematic review follows the PICO framework to guide the search process (Moher et al. 2009). The categories include:

- Population (P): Studies focusing on educational institutions, particularly those in formal or informal learning settings.
- Intervention (I): Integration of LA to study, foster, or assess creativity.
- Comparison (C): Studies comparing traditional educational methods or curricula without LA.
- Outcome (O): Insights into how LA supports creativity, including processes, outcomes, and personalized feedback.

### 2.2. Eligibility Criteria and Selection Process

Results from the database searches (search terms available in Appendix A) were uploaded to Covidence (Veritas Health Innovation 2024), where duplicates were automatically identified and removed. Titles and abstracts of each article were independently screened by all reviewers. A total of four authors participated in this initial screening stage to ensure consistency.

For articles deemed relevant during the title and abstract screening, the full texts were subsequently reviewed by two reviewers independently and disagreements were resolved through discussion or third-party adjudication. The same group of four authors completed the in-depth review process. Any discrepancies or uncertainties arising during the review process were discussed and resolved in regular team meetings.

Inclusion and exclusion criteria used to guide the selection process are detailed in Table 1. During screening, records were coded as ‘lack of focus on creativity’ when creativity was (i) peripheral to the research aims, or (ii) operationalized solely as engagement, innovation, or generic problem-solving without explicit creative constructs, tasks, or measures. This review considered studies from formal and informal learning contexts across educational levels, including K–12 (primary/secondary schooling), higher education (undergraduate and postgraduate), and adult/professional learning delivered via online platforms (such as MOOCs/LMS). Data from the included studies were extracted collaboratively by all reviewers using elicit.com. The standardized extraction template captured key elements including study design, population, sample size, findings, and limitations, ensuring consistency and accuracy.

### 2.3. Data Extraction and Management, Quality Assessment, Data Synthesis, and Reporting

Citations were managed using EndNote 21 software (The EndNote Team 2013). Titles and abstracts were screened for relevance, and eligible full texts were reviewed via Covidence, a web-based tool (Veritas Health Innovation 2024). Data extraction included study design, population, sample size, findings, and limitations and was conducted via Elicit web application (Elicit 2023). The development of the extraction protocol was informed by systematic review methodologies recommended by Gough et al. (2017).

The quality of included studies was assessed using study-design-specific tools, such as CASP (Long et al. 2020) for qualitative studies and ROBINS-I (Hasan et al. 2024) for non-randomized studies (Bown and Sutton 2010). The emphasis was on methodological rigor, clarity of reporting, and relevance to the review’s objectives.

Findings were synthesized narratively, focusing on emerging themes, variations in outcomes, and research gaps. The PRISMA guidelines were followed for reporting, and the study selection process is presented in the PRISMA flow diagram (Moher et al. 2009).

## 3. Results

**Figure 1:**
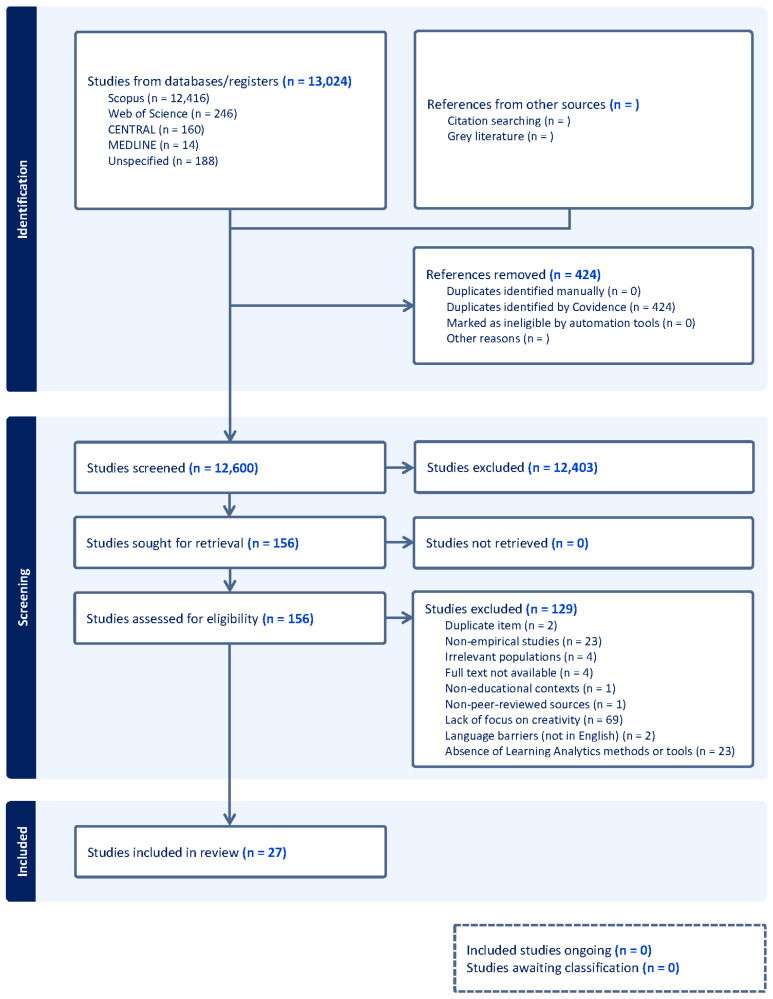
PRISMA flow diagram showing article identification, screening, eligibility, and inclusion steps. (A completed PRISMA 2020 checklist is provided in the Supplementary Materials).

A total of 12,600 unique articles were identified through database searches (Figure 1). After screening titles and abstracts, 156 full-text articles were assessed for eligibility. Of these, 129 articles were excluded based on the eligibility criteria outlined in Table 1, leaving 27 articles for inclusion in this systematic review. As shown in Figure 1, 69 of the 129 exclusions were due to the lack-of-focus-on-creativity criterion, where creativity was peripheral or conflated with engagement/innovation/general problem-solving without clear creative constructs, as detailed in Section 2.2 and Table 1.

### 3.1. Research Approach and Methodology

The majority of the research was done empirically, with four studies presenting theoretical outcomes through conceptual and review-based approaches and methodologies.

In terms of the research approach, case study and experimental study are more commonly observed types of study design. Specifically, case study design was mainly utilized for the development and validation of a learning analytics framework informed by pedagogies (Hernández-García et al. 2016; Ifenthaler and Widanapathirana 2014; Kaliisa et al. 2019; Koh et al. 2016), while experimental design, especially quasi-experimental design, was implemented for the evaluation of the creativity and innovation in learning spaces driven by LA (Saleeb 2021; Zhang et al. 2022; Yang and Ogata 2023). Other research approaches involve design-based studies examining and evaluating the effectiveness of LA-enhanced learning platforms (Charleer et al. 2016; Constapel et al. 2019), exploratory and observational-based studies targeting the use of multimodal learning analytics in project-based or collaborative learning (Spikol et al. 2018; Moon et al. 2024) and survey-based studies investigating the adoption of LA and AI capability through the lens of educators and institutions (Wang et al. 2023; El Alfy and Kehal 2024). One study done by Alexandron et al. (2019) adopted a unique replication study design to evaluate the sensitivity of the findings from two highly-cited LA MOOC studies by replicating their research settings.

In terms of the research methodology, all studies implemented either quantitative or mixed methods. There are two main groups of quantitative methods observed, including descriptive and predictive methods. Most of the studies adopted descriptive methods, such as descriptive statistics (El Alfy and Kehal 2024; Klašnja-Milićević et al. 2020; Yang and Ogata 2023), chi-square and independent sample *t*-test (Park and Kim 2022), correlation analysis (Bulut et al. 2023) and pattern analysis (Constapel et al. 2019), to derive insights from analysing the existing data. Some studies aimed to generalise the analytical results by using predictive methods built on machine learning-based models, such as First-Order Markov Model (Lahza et al. 2023), Support Vector Machines (SVM) (Ifenthaler and Widanapathirana 2014; Spikol et al. 2018) or Generalised Mixed-Effects Trees (GMET) (Fontana et al. 2021). More dynamic methods, such as social network analysis (Hernández-García et al. 2016; Kaliisa et al. 2019), text analysis (Saleeb 2021) and statistical discourse analysis (Moon et al. 2024) also attracted some attention for contextualisation of the analytical results. For mixed method studies, qualitative methods, such as interviews and focus groups (Bender and Sung 2020; Charleer et al. 2016; Koh et al. 2016), were undertaken as part of the methodology mainly for the facilitation of data collection.

### 3.2. Theoretical Framework

A significant portion of the research positions LA itself as a guiding framework, adopting a pragmatic approach where the principles of data collection, analysis, and visualization are used to measure and optimize learning processes and environments (Ifenthaler and Widanapathirana 2014; Karaoglan Yilmaz 2022; Klašnja-Milićević et al. 2020). This approach treats LA not merely as a set of tools, but as a lens through which educational phenomena are understood and improved. Several studies, however, did not identify an explicit theoretical framework, instead implying foundations in the broader ethos of educational data mining and data-driven decision-making, where the primary goal is practical improvement derived from empirical data patterns (Park and Kim 2022; Liñán and Pérez 2015; Peña-Ayala 2014).

A prominent theme is the application of theories related to student learning, cognition, and behavior. Self-Regulated Learning (SRL) is frequently used to conceptualize how LA can empower learners to manage their own progress. This is often achieved through dashboards and feedback mechanisms that make learning patterns visible, prompting students to reflect on their strategies and make adjustments (Charleer et al. 2016; Lahza et al. 2023). Similarly, theories of self-efficacy are invoked to explain how LA interventions can bolster student confidence and problem-solving skills, as personalized feedback can demystify complex topics and provide clear, actionable steps toward mastery (Karaoglan Yilmaz 2022). For group learning, frameworks like Collaborative Cognitive Load Theory (CCLT) are applied to analyze and improve team dynamics in online settings by using LA to identify points of confusion or information overload within discussions (Zhang et al. 2022). Meanwhile, Social Learning Analytics (SLA) and Social Network Analysis (SNA) are used to conceptualize learning as a socially mediated process, where visualizing interaction networks can reveal key influencers, isolated students, and the overall health of a learning community (Kaliisa et al. 2019; Hernández-García et al. 2016). Other learning-centric theories, such as constructivism and deep learning, are used to evaluate whether student-centered activities in innovative virtual learning spaces are achieving their intended outcomes (Saleeb 2021), while experiential and situated learning provide a basis for developing multimodal LA systems that capture learning as it happens in rich, authentic contexts (Spikol et al. 2018).

Several studies incorporate established technological and behavioral models to understand the human factors surrounding LA adoption. The Unified Theory of Acceptance and Use of Technology (UTAUT), along with its predecessors like the Theory of Reasoned Action (TRA) and Behavioral Reasoning Theory (BRT), provides a lens for examining the factors influencing educators’ adoption of LA tools, such as their perceptions of the tools’ usefulness (performance expectancy) and ease of use (effort expectancy) (El Alfy and Kehal 2024). In the context of artificial intelligence, Resource-Based Theory (RBT) is used to conceptualize how an institution’s AI capabilities function as a strategic asset, with LA serving as the mechanism to measure the impact of that asset on student outcomes like creativity and learning performance (Wang et al. 2023).

Finally, some research is grounded in specific educational or analytical approaches that connect directly to LA’s function. For instance, Cognitivist Media Theory (CMT) is applied to understand audience responses to creative media, with LA and biometrics offering an objective, data-driven window into engagement that goes beyond subjective reports (Bender and Sung 2020). In parallel, frameworks for formative assessment are used as a foundation for LA models that analyze collaborative dynamics and predict student performance. Here, LA operationalizes the principles of formative assessment at scale, transforming data from continuous, low-stakes activities into powerful, predictive insights that can guide timely interventions (Bulut et al. 2023; Moon et al. 2024).

### 3.3. LA Tools and Techniques

The literature reviewed here demonstrates a comprehensive picture of how LA tools and techniques are being used in a wide range of educational settings. In general, the LA tools and techniques can be categorised into statistical and machine learning-based techniques, visualization and dashboards, multimodal LA systems, personalized learning systems and comprehensive LA frameworks.

The majority of the research implemented statistical and machine learning-based LA techniques, including text analysis and Natural Language Processing (NLP), predictive modeling, clustering and pattern recognition, SNA and statistical and data mining. Text analysis and NLP techniques, such as Jieba, GloVe, and Coh-Metrix, were used to analyze discourse and linguistic features from discussion transcripts to assess collaborative states and language quality (Zhang et al. 2022; Kaliisa et al. 2019). Predictive modeling approaches, such as SVM, Bayesian networks, neural networks, logistic regression, and tree-based models like GMET, were applied to predict student performance, dropout risk, and provide personalized recommendations using LMS data and demographic information (Klašnja-Milićević et al. 2020; Ifenthaler and Widanapathirana 2014; Spikol et al. 2018; Yan et al. 2021; Fontana et al. 2021; Bulut et al. 2023). Clustering and pattern recognition methods, such as k-means clustering, Bayes theorem and hidden Markov models, were employed with tools like TraMineR and pMineR to detect learning tactics, sequence patterns, and behavioral strategies from platform trace data (Peña-Ayala et al. 2017; Lahza et al. 2023; Peña-Ayala 2014). SNA was conducted using NodeXL and Gephi to visualize and analyze student interactions, collaborative activities, and social learning processes from LMS logs and discussion forums (Kaliisa et al. 2019; Hernández-García et al. 2016; Hernández-García and Conde 2014). Statistical and data mining techniques, such as chi-square tests, Item Response Theory (IRT), Partial Least Squares Structural Equation Modeling (PLS-SEM), and causal inference analysis, were performed to examine relationships between learning behaviors, strategies, and performance outcomes (Park and Kim 2022; Alexandron et al. 2019; Wang et al. 2023; Constapel et al. 2019).

Some studies utilized visualization and dashboard tools, including D3.js, Processing.js, StepUp!, radar charts, and learning dashboards, to increase the interpretability of the analytical results through displaying learning efforts, teamwork competencies, student engagement patterns, and performance metrics from diverse data sources involving RSS feeds, time logs, and LMS data (Koh et al. 2016; Santos et al. 2013; Charleer et al. 2016; Saleeb 2021).

Multimodal LA systems and personalized learning systems are more sophisticated LA tools used in recent studies. While multimodal LA systems integrated eye-tracking systems, facial expression analysis tools like Noldus FaceReader and OpenFace 2.0, audio transcription services, and sensor data to analyze emotional states, collaboration patterns, and physiological responses during learning activities (Bender and Sung 2020; Moon et al. 2024; Spikol et al. 2018), personalized learning systems focused more on the identification of engagement patterns to provide actionable feedback and deliver tailored support based on historical learning data using generic educational data mining techniques (Yang and Ogata 2023; Karaoglan Yilmaz 2022).

Particularly, Liñán and Pérez (2015) developed a comprehensive LA framework encompassed multiple methods including prediction, clustering, relationship mining, sentiment analysis, discourse analysis, and sense-making models to analyze student performance and interaction data across various educational contexts.

In summary, the analyzed literature reveals a comprehensive and evolving landscape of a wide range of LA tools and techniques aimed at enhancing educational outcomes. These methodologies range from advanced machine learning for prediction and personalization, sophisticated data mining for uncovering patterns, and various approaches to social and multimodal analytics for understanding complex interactions. The consistent utilization of various software applications and analytical frameworks demonstrates a collective effort towards benefiting learner data to improve pedagogical practices and a deeper understanding of the learning process across different educational contexts.

### 3.4. Creativity Assessment Framework and Metrics

Creativity in educational contexts is multifaceted and difficult to assess due to its dynamic, context-dependent, and multidimensional nature (Long et al. 2022; Bolden et al. 2020). In light of this complexity, our analysis identifies three overlapping domains essential for assessment frameworks: divergent thinking, creative product evaluation, and behavioral/process-based creativity metrics. Accordingly, we included an ancillary corpus of 23 foundational works on creativity assessment to serve as analytic scaffolds, defining constructs, criteria, and validity evidence against which we compared LA-derived measures. These sources cover established divergent-thinking instruments, product-based consensual evaluation approaches, and emerging behavioral/process metrics. They function as frameworks rather than LA interventions and are therefore reported separately from the 27 LA studies.

One widely recognized method is the divergent thinking assessment, which evaluates the fluency, originality, flexibility, and elaboration of student responses to open-ended prompts. Tools such as the Torrance Tests of Creative Thinking (TTCT) (Plucker 1999) and their adaptations remain central, particularly in measuring individual idea generation capabilities. More recently, adaptations like the Rasch-based validation for elementary creativity (Noorkholisoh et al. 2024) and automated TTCT scoring via machine learning (Cropley et al. 2024) have enhanced scalability and objectivity.

Several studies advocate for the Consensual Assessment Technique (CAT), where creativity is judged by experts on the quality of student artefacts (Hadas and Hershkovitz 2025; McKenna et al. 2013). While robust, CAT’s reliance on expert judgment can present challenges for standardization and scalability. Recent work suggests integrating CAT with AI-based tools to enhance consistency across evaluators (Kovalkov et al. 2021).

Behavioral and process-based approaches offer promising alternatives. For example, observational tools like the Creative Collaboration Scale (CCS) (Mavri et al. 2020) or process mining techniques (Israel-Fishelson and Hershkovitz 2024) capture real-time indicators such as interaction patterns, problem-solving sequences, and biometric markers of engagement. These tools align with newer frameworks that prioritize process over output in assessing creativity (Kupers et al. 2018; Ryu et al. 2024).

AI-driven and multimodal learning environments have also introduced automatic creativity detection frameworks. Kovalkov et al. (2020) demonstrate the feasibility of modelling creativity in Scratch programming using visual, auditory, and behavioral signals. Similarly, assessment tools have emerged that mine log data to evaluate problem-solving diversity (Olivares-Rodríguez et al. 2017) or use biometric data to infer cognitive and affective states during creative tasks (Ryu et al. 2024).

Some frameworks focus on the disciplinary context. In engineering, creativity is operationalized via solution novelty and feasibility, often measured using the Engineering Creativity Assessment Tool (ECAT) (Akdemir-Beveridge et al. 2025) or task-specific rubrics (Denson et al. 2015). In computing, rubrics may include functionality, aesthetics, and innovation criteria (da Cruz Alves et al. 2021). Similarly, in screen production, tools like gaze tracking and emotion recognition software assess how creative choices impact audiences (Bender and Sung 2020).

Emerging approaches also embrace socio-cultural and collaborative models of creativity. Tan et al. (2014) propose dialogic frameworks for collective creativity, while tools like the TCD-D app (De Lorenzo et al. 2023) allow students to reflect on and evaluate their own creative processes. These perspectives align with contemporary pedagogical shifts toward constructivist, inquiry-based, and socially mediated learning environments.

Additionally, psychometric validation of instruments is increasingly common. Studies have used structural equation modelling (Wang et al. 2023), Rasch analysis (Noorkholisoh et al. 2024), and bootstrapping techniques to confirm the reliability and validity of creative thinking assessments across age groups and disciplines.

Importantly, creative assessment should be responsive to technological and cultural shifts. Several scholars highlight the importance of contextually sensitive and inclusive tools that account for learners’ backgrounds, disciplinary norms, and learning settings (Zaremohzzabieh et al. 2025; Smyrnaiou et al. 2020). Creativity in online learning, for example, may manifest differently than in traditional settings, requiring novel data sources and frameworks.

Finally, creativity assessment must evolve alongside educational technologies. As generative AI becomes embedded in learning environments, frameworks must distinguish between human and AI-generated creativity (Hadas and Hershkovitz 2025). Future systems may integrate AI co-assessment and feedback to scaffold creative development while preserving authenticity and learner agency.

### 3.5. Findings from the Studies

A total of 50 studies were included in this review, combining 27 Learning Analytics (LA) studies with 23 additional works focused on creativity assessment frameworks and metrics (this section synthesizes the 27 LA studies, while the 23 assessment works in Section 3.4 serve as frameworks and are not counted as LA interventions). These studies highlight the diverse applications of LA and creativity research across various educational contexts, showcasing methods to analyze, predict, and improve learning processes. The key findings are summarized as follows:

1. Enhanced Predictive Analytics: Predictive modeling techniques—such as machine learning, clustering, and regression—were used to identify at-risk students, model learner profiles, and optimize personalized support (Fontana et al. 2021; Peña-Ayala et al. 2017). Tools like SVM and Bayesian Knowledge Tracing (BKT) offered validated mechanisms for profiling and prediction (Ifenthaler and Widanapathirana 2014; Yan et al. 2021).
2. Collaboration and Teamwork Dynamics: Several studies employed multimodal LA (MMLA) techniques such as statistical discourse analysis, gaze tracking, facial expression recognition, and peer ratings to understand group interaction patterns and enhance collaborative competencies (Koh et al. 2016; Moon et al. 2024). Creativity in group contexts was further examined using frameworks like the Assessment Scale for Creative Collaboration (Mavri et al. 2020).
3. Technological Integration and Personalized Learning: Adaptive dashboards and personalized analytics interventions—such as BookRoll and face-tracking systems—helped deliver real-time feedback and increased behavioral engagement (Yang and Ogata 2023; Moon et al. 2024). Intelligent systems that tailored content based on learning profiles were positively associated with creative thinking (Wang et al. 2023).
4. Learning Design and Visualization: Dashboards and data visualization tools like radar charts, scatterplots, and heatmaps were commonly used to facilitate metacognition and learner reflection (Charleer et al. 2016; Hernández-García et al. 2016). Analytics-enabled platforms like StepUp! supported self-regulated learning through time tracking and artefact production (Santos et al. 2013).
5. Creativity Metrics and Assessment Approaches: Emerging approaches to assess creativity include:
  - Product-based creativity metrics: Tools such as the Test of Creative Thinking–Drawing Production (TCT-DP) were used to automatically assess fluency, elaboration, and originality in outputs (Cropley et al. 2024).
  - Behavioral and system-logged metrics: Query diversity (Olivares-Rodríguez et al. 2017), Scratch-based code complexity (Kovalkov et al. 2021), and biometric responses (Bender and Sung 2020) were explored as proxies for creative performance.
  - Framework-based surveys and scales: Studies employed structured creativity assessment frameworks across domains like STEAM, engineering, and narrative writing (Bolden et al. 2020; Akdemir-Beveridge et al. 2025).
6. AI-Supported Creativity Assessment: New research demonstrates how generative AI models, Natural Language Processing (NLP), and automatic scoring can assess creative ideas and processes across interventions (Hadas and Hershkovitz 2025; Marrone and Cropley 2022). These methods offer scalability but still require careful validation to ensure construct accuracy.

#### Challenges

While progress has been made, key challenges persist in applying creativity assessment within LA environments:

- Definitional Ambiguity: Variability in the conceptualization of creativity (e.g., product- vs. process-oriented; domain-general vs. domain-specific) remains a major obstacle to standardized measurement (Henriksen et al. 2021; Bolden et al. 2020).
- Methodological Limitations: Many instruments lack robust validation across different educational contexts or fail to accommodate domain-specific demands. The scalability of creativity assessments, especially in online or automated environments, remains limited (Kovalkov et al. 2021; Wang et al. 2023).
- Ethical and Technological Barriers: Real-time monitoring through LA dashboards and biometric sensors raises ethical concerns regarding student consent, data privacy, and algorithmic bias (Hernández-García et al. 2016; Hadas and Hershkovitz 2025).
- Over-reliance on Self-report Instruments: While tools such as the Engineering Creativity Assessment Tool (Akdemir-Beveridge et al. 2025) and STEAM-based creativity scales (Yulianti et al. 2024) are widely used, they depend on subjective measures that may not fully capture creativity in action.

## 4. Discussion, Future Directions, and Conclusion

This systematic review highlights the transformative potential of Learning Analytics (LA) in educational contexts, emphasizing its significant role in personalized interventions, predictive assessments, and actionable feedback mechanisms. Drawing on the foundational definition provided by Siemens and Baker (2012), LA involves measuring, collecting, analyzing, and reporting data to enhance learning processes and outcomes. The evidence underscores LA’s capability to support self-efficacy, promote creativity, and increase learner engagement through data-driven insights and targeted pedagogical interventions.

### 4.1. Self-Efficacy and Learning Analytics

Self-efficacy, as articulated by (Bandura et al. 1999), has been identified as a pivotal construct within LA implementations. Higher self-efficacy levels correlate positively with increased learner engagement and more effective utilization of analytics-driven tools. Multimodal learning analytics (MMLA), for example, have been shown to bolster learners’ collaborative skills and confidence, ultimately leading to improved educational outcomes (Koh et al. 2016; Moon et al. 2024). Similar outcomes were observed through creativity-oriented interventions, where assessments aimed at enhancing students’ creative confidence contributed positively to their overall academic self-efficacy (Yulianti et al. 2024).

### 4.2. Methodological Diversity and Innovations

The reviewed literature reveals significant methodological diversity and innovation within LA. Approaches ranging from statistical methods, predictive analytics, visualization and dashboards tools to adaptive and intelligent systems underscore LA’s broad applicability and technological sophistication. Advanced techniques such as emotion-detection platforms like OpenFace, and audio transcription services exemplify this cutting-edge innovation (Bender and Sung 2020; Moon et al. 2024; Ifenthaler and Widanapathirana 2014; Yan et al. 2021). Despite these advancements, existing studies focused more on the LA interventions on student engagement and performance through physical and digital traces, and the absence of standardized frameworks restricts the scalability and comparability of LA interventions across diverse educational environments (Ochoa et al. 2017). To address this, researchers have advocated for more rigorous, consistent methodological frameworks for LA-informed teaching pedagogies and practices, particularly in creativity assessment (Cropley et al. 2024).

### 4.3. Creativity in Learning Analytics

Although creativity has traditionally been indirectly addressed within LA literature, recent studies have increasingly employed explicit creativity assessments. Metrics involving product-oriented tools, such as the Test of Creative Thinking–Drawing Production (TCT-DP), provide robust automated approaches to creativity measurement, capturing dimensions like originality and elaboration effectively (Cropley et al. 2024). Additionally, behavioral analytics, including biometrics such as eye tracking and facial expression analysis, offer powerful means of assessing real-time creative engagement and responses (Bender and Sung 2020). Nonetheless, the literature reveals persistent methodological inadequacies of reliance on small participation samples affecting the validity and generalizability of the studies and highlights ongoing gaps in standardized and scalable creativity assessment frameworks, emphasizing the need for clearer definitions and universally applicable metrics (Bolden et al. 2020; Long et al. 2022).

### 4.4. Evidence-Based Decision-Making

LA dashboards and visualization tools have significantly enhanced educational practices by enabling educators and learners to make informed, evidence-based decisions. Tools like StepUp! and radar charts facilitate real-time feedback and encourage self-regulation and reflective learning (Charleer et al. 2016; Santos et al. 2013). The integration of advanced visual analytics not only clarifies complex learning patterns but also significantly improves learner and educator responsiveness, thus enabling tailored pedagogical adjustments (Hernández-García and Conde 2014).

### 4.5. Conclusion and Future Directions

Learning Analytics holds immense promise in revolutionizing educational practices by promoting innovation, engagement, and adaptive learning. To maximize its transformative potential, future research and practical implementations should prioritize the following strategic directions:

#### 4.5.1. Standardizing Creativity Metrics

Robust frameworks such as the 4 Ps of Creativity (Person, Process, Product, Press) and validated instruments like the ECAT must be consistently integrated across educational contexts to ensure comparability and reliability of creativity assessments (Bolden et al. 2020; Akdemir-Beveridge et al. 2025). For example, the 4 Ps of Creativity framework can be implemented in the course rubrics and the ECAT instrument can be deployed with standardized scoring protocols as part of the assessments.

#### 4.5.2. Expanding Multimodal and AI-Supported Analytics

The use of advanced multimodal technologies, including biometric analyses and generative AI models, should be scaled and rigorously validated across educational settings with controlled comparison groups, documented performance evaluations and reproducible results. Such technologies offer promising methods for capturing the complex, multifaceted nature of creativity in real-time educational scenarios (Moon et al. 2024; Hadas and Hershkovitz 2025; Ryu et al. 2024).

#### 4.5.3. Addressing Ethical and Privacy Concerns

Ethical considerations such as privacy, consent, and equity must be rigorously addressed to establish trust in LA practices. Ensuring transparent data usage policies and equitable access to analytics tools and establishing institutional review boards specifically for LA that include student representatives and community stakeholders remains paramount for sustainable LA implementation (Hernández-García and Conde 2014).

#### 4.5.4. Enhancing Scalability and Validation

To facilitate broad adoption, analytics tools such as Scratch-based creativity assessments and automated scoring systems need systematic validation and adaptation to diverse educational contexts. Scalable solutions are critical to ensure widespread applicability and reliability, for instance, testing Scratch-based creativity assessments with diverse student cohorts with proved reliability coefficients and evidence of predictive validity against creativity outcomes (Kovalkov et al. 2021; Cropley et al. 2024).

#### 4.5.5. Integrating Creativity Explicitly into Learning Analytics

Recognizing creativity explicitly as a measurable and integral construct within LA frameworks is essential to foster a deeper understanding and support of innovative and adaptive learning outcomes. Comprehensive integration of creativity assessment approaches can significantly enhance LA’s educational impact. LMS should add creativity as a trackable competency indicator alongside traditional metrics, with automated alerts when students demonstrate creative breakthroughs or prolonged stagnation, enabling instructors to provide timely and targeted feedback (Marrone and Cropley 2022).

#### 4.5.6. Bolstering Self-Efficacy Through Targeted Interventions

Strategic emphasis on developing learners’ self-efficacy should continue to guide LA tool development and deployment, such as personalized visualizations demonstrating students’ creative growth over time and an award mechanism for creative progression. Integrative approaches that simultaneously address cognitive and affective aspects of learning are essential to fully realize the educational potential of LA (Bandura et al. 1999; Yulianti et al. 2024).

Ultimately, Learning Analytics demonstrates significant potential to revolutionize educational landscapes by supporting personalized learning experiences, innovative creativity assessments, and evidence-based pedagogical decisions. By addressing identified methodological and ethical challenges, future research can more effectively harness LA’s capabilities to foster comprehensive, inclusive, and adaptive educational environments.

## Figures and Tables

**Table 1:** Inclusion and exclusion criteria.

Criteria	Inclusion	Exclusion
Population	Studies conducted in formal or informal educational settings, spanning K–12 (primary/secondary), higher education (undergraduate/postgraduate), and adult/professional learning, including schools, universities, and online learning platforms.	Studies conducted in non-educational contexts, such as business, healthcare, or non-academic organizations.
Intervention/Exposure	Studies explicitly exploring the application or development of LA tools or frameworks in educational contexts.	Studies not addressing the use of LA or unrelated to its application in education.
Outcome	Research examining creativity, including fostering creative thinking, capturing creative processes, or providing creativity-oriented feedback.	Studies not focusing on creativity, or where creativity is tangential to the primary research goals (i.e., creativity was treated peripherally or operationalized as engagement, innovation, or general problem-solving without clear creative constructs).
Study Design	Peer-reviewed journal articles, conference papers, book chapters, systematic reviews, or meta-analyses.	Non-peer-reviewed materials, such as blog posts, editorials, or unpublished dissertations.
Methodological Rigor	Studies employing robust quantitative, qualitative, or mixed methods with clearly defined and reproducible methodologies.	Studies lacking methodological rigor, transparency, or sufficient data to support their conclusions.
Publication Date	Articles published between September 2012 and September 2024.	Articles published before September 2012.
Language	Publications available in English.	Non-English publications without an available translation.
Full-Text Accessibility	Studies with full-text articles accessible for review.	Studies with inaccessible or unavailable full text.

## Data Availability

No new data were created or analyzed in this study.

## Supplementary Materials

The following supporting information can be downloaded at: https://www.mdpi.com/article/10.3390/jintelligence13120153/s1, Table S1: PRISMA 2020 checklist (Page et al. 2021).

## Appendix A

**Table A1:** Search Term for the Systematic Literature Review.

Population Terms	Exposure Terms 1	Exposure Terms 2	Exposure Term 3
“distance education” OR “digital education*” OR “online learning*” OR “virtual class*”??	“learning analytic*” OR “learning metric*” OR “data analysis”	creativ* OR “creative thinking” OR “creative pedagogy” OR pedagog* OR innovativ*	“student engagement” OR “educational assessment” OR “academic performance” OR “learning outcomes”

**Table A2:** Example Search Strategy.

Database	Syntax	Limits
Web of Science	TS = (“distance education” OR “digital education*” OR “online learning*” OR “virtual class*”) AND TS = (“learning analytic*” OR “learning metric*” OR “data analysis”) AND TS = (creativ* OR “creative thinking” OR “creative pedagogy” OR pedagog* OR innovativ*) AND TS = (“student engagement” OR “educational assessment” OR “academic performance” OR “learning outcomes”)	1 September 2012 onwards English language
