# Supplementary material for: Creativity in Learning Analytics: A Systematic Literature Review
Source: J Intell. 2025 Nov 23;13(12):153. doi: 10.3390/jintelligence13120153 (PMC12733823; doi:10.3390/jintelligence13120153)
Supplement: Supplementary file 1 [file jintelligence-13-00153-s001.zip › jintelligence-3892465-Supplementary Materials.pdf]

PRISMA 2020 Checklist

Section and Topic	Item #	Checklist item	Location where item is reported
**TITLE**			
Title	1	Identify the report as a systematic review.	Reported in Title: Creativity in Learning Analytics: A Systematic Literature Review
**ABSTRACT**			
Abstract	2	See the PRISMA 2020 for Abstracts checklist.	Abstract structured to include background, objectives, methods (databases searched, eligibility), results (27+ studies), conclusions, and implications.
**INTRODUCTION**			
Rationale	3	Describe the rationale for the review in the context of existing knowledge.	Importance of creativity as a 21st-century skill (OECD 2018, Avenckuté 2020, Henriksen 2021), and rationale for systematically reviewing its integration with LA.
Objectives	4	Provide an explicit statement of the objective(s) or question(s) the review addresses.	Explicit research questions stated in Methods (RQ1–RQ4).
**METHODS**			
Eligibility criteria	5	Specify the inclusion and exclusion criteria for the review and how studies were grouped for the syntheses.	eported in Methods 2.2 (Inclusion/Exclusion criteria table).
Information sources	6	Specify all databases, registers, websites, organisations, reference lists and other sources searched or consulted to identify studies. Specify the date when each source was last searched or consulted.	Reported in Methods 2.2: Databases searched include Scopus, Web of Science, ERIC, ProQuest, and

PRISMA 2020 Checklist

Section and Topic	Item #	Checklist item	Location where item is reported
			Google Scholar, with September 2012–September 2024 coverage.
Search strategy	7	Present the full search strategies for all databases, registers and websites, including any filters and limits used.	Search terms in Appendix 1 (provided in manuscript).
Selection process	8	Specify the methods used to decide whether a study met the inclusion criteria of the review, including how many reviewers screened each record and each report retrieved, whether they worked independently, and if applicable, details of automation tools used in the process.	Reported in Methods 2.2: Titles/abstracts screened independently by 4 authors using Covidence, conflicts resolved by discussion or third reviewer.
Data collection process	9	Specify the methods used to collect data from reports, including how many reviewers collected data from each report, whether they worked independently, any processes for obtaining or confirming data from study investigators, and if applicable, details of automation tools used in the process.	Reported in Methods 2.3: Data extracted collaboratively via Elicit.com with standardized template; Covidence for review management.
Data items	10a	List and define all outcomes for which data were sought. Specify whether all results that were compatible with each outcome domain in each study were sought (e.g. for all measures, time points, analyses), and if not, the methods used to decide which results to collect.	Reported in Methods 2.3: Outcomes include creativity processes, measures, feedback, frameworks, and limitations.
	10b	List and define all other variables for which data were sought (e.g. participant and intervention characteristics, funding sources). Describe any assumptions made about any missing or unclear information.	Population, study design, sample size, LA tools, creativity metrics.
Study risk of bias assessment	11	Specify the methods used to assess risk of bias in the included studies, including details of the tool(s) used, how many reviewers assessed each study and whether they worked independently, and if applicable, details of automation tools used in the process.	Reported in Methods 2.3: CASP for qualitative studies (Long et al.,

Section and Topic	Item #	Checklist item	Location where item is reported
			2020), ROBINS-I for non-randomized (Hasan et al., 2024).
Effect measures	12	Specify for each outcome the effect measure(s) (e.g. risk ratio, mean difference) used in the synthesis or presentation of results.	Not applicable (narrative synthesis).
Synthesis methods	13a	Describe the processes used to decide which studies were eligible for each synthesis (e.g. tabulating the study intervention characteristics and comparing against the planned groups for each synthesis (item #5)).	Reported in Methods 2.3: Narrative synthesis guided by PRISMA; thematic grouping into predictive analytics, collaboration, visualization, creativity frameworks.
	13b	Describe any methods required to prepare the data for presentation or synthesis, such as handling of missing summary statistics, or data conversions.	Same as 13a response.
	13c	Describe any methods used to tabulate or visually display results of individual studies and syntheses.	Same as 13a response.
	13d	Describe any methods used to synthesize results and provide a rationale for the choice(s). If meta-analysis was performed, describe the model(s), method(s) to identify the presence and extent of statistical heterogeneity, and software package(s) used.	Same as 13a response.
	13e	Describe any methods used to explore possible causes of heterogeneity among study results (e.g. subgroup analysis, meta-regression).	Same as 13a response.
	13f	Describe any sensitivity analyses conducted to assess robustness of the synthesized results.	Same as 13a response.
Reporting bias assessment	14	Describe any methods used to assess risk of bias due to missing results in a synthesis (arising from reporting biases).	Discussed in 3.6.1 Challenges (publication bias and methodological gaps).
Certainty assessment	15	Describe any methods used to assess certainty (or confidence) in the body of evidence for an outcome.	Addressed narratively in Discussion (limitations in evidence, methodological rigor).
**RESULTS**			
Study selection	16a	Describe the results of the search and selection process, from the number of records identified in the search to the number of studies included in the review, ideally using a flow diagram.	Reported in Results: 12,600 →

Section and Topic	Item #	Checklist item	Location where item is reported
			156 full texts → 27 included; PRISMA flow diagram in Figure 1.
	16b	Cite studies that might appear to meet the inclusion criteria, but which were excluded, and explain why they were excluded.	Reasons summarized in PRISMA diagram and text (eligibility criteria applied).
Study characteristics	17	Cite each included study and present its characteristics.	Summarized in Table 2.
Risk of bias in studies	18	Present assessments of risk of bias for each included study.	Quality assessments performed (CASP, ROBINS-I).
Results of individual studies	19	For all outcomes, present, for each study: (a) summary statistics for each group (where appropriate) and (b) an effect estimate and its precision (e.g. confidence/credible interval), ideally using structured tables or plots.	Reported in Section 3 (summaries and citations).
Results of syntheses	20a	For each synthesis, briefly summarise the characteristics and risk of bias among contributing studies.	Grouped by themes (predictive analytics, collaboration, personalization, visualization, creativity frameworks). No meta-analysis.
	20b	Present results of all statistical syntheses conducted. If meta-analysis was done, present for each the summary estimate and its precision (e.g. confidence/credible interval) and measures of statistical heterogeneity. If comparing groups, describe the direction of the effect.	Grouped by themes (predictive analytics, collaboration, personalization, visualization, creativity frameworks). No meta-analysis.
	20c	Present results of all investigations of possible causes of heterogeneity among study results.	Grouped by themes (predictive analytics, collaboration, personalization, visualization, creativity frameworks). No

PRISMA

PRISMA 2020 Checklist

Section and Topic	Item #	Checklist item	Location where item is reported
			meta-analysis.
	20d	Present results of all sensitivity analyses conducted to assess the robustness of the synthesized results.	Grouped by themes (predictive analytics, collaboration, personalization, visualization, creativity frameworks). No meta-analysis.
Reporting biases	21	Present assessments of risk of bias due to missing results (arising from reporting biases) for each synthesis assessed.	Acknowledged in Challenges (limited frameworks, definitional ambiguity).
Certainty of evidence	22	Present assessments of certainty (or confidence) in the body of evidence for each outcome assessed.	Reported narratively in Discussion.
**DISCUSSION**			
Discussion	23a	Provide a general interpretation of the results in the context of other evidence.	Reported in Section 4.
	23b	Discuss any limitations of the evidence included in the review.	Reported in Discussion.
	23c	Discuss any limitations of the review processes used.	Noted: publication bias, exclusion of non-English, scope limited to 2012–2024.
	23d	Discuss implications of the results for practice, policy, and future research.	Recommendations for practice and future research in Section 4.
**OTHER INFORMATION**			
Registration and protocol	24a	Provide registration information for the review, including register name and registration number, or state that the review was not registered.	This review was not registered
	24b	Indicate where the review protocol can be accessed, or state that a protocol was not prepared.	Not prepared.
	24c	Describe and explain any amendments to information provided at registration or in the protocol.	None
Support	25	Describe sources of financial or non-financial support for the review, and the role of the funders or sponsors in the review.	No external funding; internal academic collaboration.
Competing	26	Declare any competing interests of review authors.	None

PRISMA 2020 Checklist

Section and Topic	Item #	Checklist item	Location where item is reported
interests			
Availability of data, code and other materials	27	Report which of the following are publicly available and where they can be found: template data collection forms; data extracted from included studies; data used for all analyses; analytic code; any other materials used in the review.	Data extraction tables and materials available upon request.

Table S1: PRISMA 2020 checklist

From: Page MJ, McKenzie JE, Bossuyt PM, Boutron I, Hoffmann TC, Mulrow CD, et al. The PRISMA 2020 statement: an updated guideline for reporting systematic reviews. BMJ 2021;372:n71. doi: 10.1136/bmj.n71. This work is licensed under CC BY 4.0. To view a copy of this license, visit https://creativecommons.org/licenses/by/4.0/
